# CHI3L1 promotes tumor progression by activating TGF-β signaling pathway in hepatocellular carcinoma

**DOI:** 10.1038/s41598-018-33239-8

**Published:** 2018-10-09

**Authors:** Qing-Chong Qiu, Lin Wang, Shan-Shan Jin, Guan-Feng Liu, Jie Liu, Liang Ma, Rui-Fang Mao, Ying-Ying Ma, Na Zhao, Ming Chen, Biao-Yang Lin

**Affiliations:** 1 College of life science, Zhejiang University, Hangzhou, P. R. China; 2 Collaborative Innovation Center for Diagnosis and Treatment of Infectious Diseases, The First Affiliated Hospital, School of Medicine, Zhejiang University, Hangzhou, China; 3 Systems Biology Division, Zhejiang-California International Nanosystems Institute (ZCNI), Zhejiang University, Hangzhou, Zhejiang Province P. R. China; 4 Department of Urology, University of Washington, Seattle, USA

## Abstract

CHI3L1 (YKL40) is a secreted glycoprotein and elevated serum CHI3L1 level has been proved to be associated with poor prognosis in many human cancers. However, the mechanism of how CHI3L1 causes poor prognosis in cancers is still unknown. Here, considering that CHI3L1 is a liver specific/enriched protein, we use hepatocellular carcinoma as a model to study the function of CHI3L1. We showed that, both *in vivo* and *in vitro*, overexpression of CHI3L1 could promote liver cancer cells growth, migration and invasion. We then used RNA-seq to analyze the expression profiles of CHI3L1 overexpressed in two HCC cell lines and found that CHI3L1 overexpression affected genes that were involved in cell-cell adhesion, extracellular exosome and adherens junction. Western blot analysis further revealed that CHI3L1 could activate TGF-β signal pathways. Our data added new understanding of the mechanism of CHI3L1’s action. 1) CHI3L1 promoted cancer cell proliferation by regulating cell cycles; 2) CHI3L1 promoted cancer cell invasion and metastasis; 3) CHI3L1 regulate liver cancer potentially by regulating the TGF-β signaling pathways; 4) CHI3L1 has direct kinase activities or activate kinase to phosphorylate SMAD2, SMAD3.

## Introduction

Hepatocellular carcinoma (HCC) is the second leading cause of cancer death in men in China and there are more than 500,000 new cases diagnosed yearly^1^. Chronic hepatitis to liver cirrhosis and to hepatocellular carcinoma (HCC) is the typical path for HCC although some patients developed into HCC without cirrhosis. The most important character of liver cancer is its close association with liver fibrosis, with more than 80% of HCC developed from fibrotic livers^2^. Toxins, alcohol abuse, fatty liver and virus infection all lead to liver fibrosis^3^.

CHI3L1 (also known as chitinase 3-like 1 or human cartilage glycoprotein-39) encodes a 40 KDa secreted glycoprotein. Although it contains a chitin-binding domain (DXXDXDXE), it lacks the chitinase activity due to variations in the D and E amino acid residues at position D138 and E140^4^. CHI3L1 is secreted by primary immune cells, endothelial cells, inflammatory cells and cancer cells. As an inflammatory mediator, CHI3L1 could be activated by various cytokines, including IL6, TNF-a, IL18, and elevated CHI3L1 level could promote inflammatory function and tissue remodeling^5,6^. Furthermore, higher serum CHI3L1 levels are associated with worse prognosis and poor survival in several cancers^7–9^.

We have previously showed that serum CHI3L1 levels increased with stages of liver fibrosis, and is a good biomarker of substantial fibrosis^10^. We also find the expression of CHI3L1 at both the protein and RNA levels is higher in liver cancer tissues than those in the normal liver tissues using both proteomic and transcriptomic analysis^11^. Pan *et al*. showed that CHI3L1 is increased in HCC cells compared to tumor-adjacent cells and further increased in metastatic HCC^12^. We hypothesize that CHI3L1 promotes cell proliferation and migration in liver cancer cells. In this study, we conducted a functional study of CHI3L1 both *in vivo* and *in vitro*. We analyzed expression profiles caused by CHI3L1 overexpression using RNA-seq. Finally, we showed that that CHI3L1 could activate TGF-β signal pathways at protein levels by Western blot analysis. This study demonstrated for the first time the roles of CHI3L1 in liver cancer carcinogenesis and suggested that CHI3L1 was a potential target for developing liver cancer therapy.

## Materials and Methods

### Construction of the overexpression and knockdown construct of CHI3L1

CHI3L1 gene (NM_001276.2) was amplified from the CHI3L1 cDNA ORF clone (Sino Biological, Shanghai, China). Then full-length human CHI3L1 cDNA was cloned into the pcDNA3.1 vector (Invitrogen, California, USA). Green fluorescent protein (GFP) was cloned into the same pcDNA3.1 vector to generate the control vector. Three oligonucleotide sequences, CHI3L1-shRNA1, CHI3L1-shRNA2, CHI3L1-shRNA3 and non-target shRNA (the sequences were showed in the supplementary materials) were cloned into the pSilencer4.1 plasmid to generate the CHI3L1 knockdown and the control vectors.

### Human cell culture and establishment of cell lines

Human liver cancer cell lines, Bel7404, HCCLM3, MHCC97L, HepG2.2.215 and HepG2 were purchased from ATCC. Cells were cultured in RPMI 1640 or DMEM supplemented with 10% FBS, 1% streptomycin and amphotericin B. All cell lines were cultured in 5% CO_2_ incubator at 37 °C.

Cells were seeded into 6-wells plates one day before transfection. Endofectin™-plus (Genecopoeia, Rockville, USA) was used to transfect the cells when the confluence of cells reached 80–90% using the transfection methods provided by the reagent supplier. Cells were harvested and reseeded into 100 mm plates one day after transfection. Geneticin and hygromycin was used to select for the drug-resistant cells and western blot was performed to confirm the positive cell clone.

### Immunofluorescence assay

The cells were fixed with 4% formaldehyde for 10 min, and then incubated in 1% BSA for 1 hour to permeabilize the cells and to block non-specific protein-protein interactions. The cells were then incubated with the rabbit-antihuman CHI3L1 antibody ab77528 (Abcam, Shanghai, China) at 5 μg/ml overnight at 4 °C. The secondary antibody, goat anti-rabbit lgG conjugating to FITC (Sangon Biotech, Shanghai, China) was used at the dilution of 1:1000 at room temperature for 1 hour. DAPI (Sangon Biotech, Shanghai, China) was used to stain the cell nuclei at the concentration of 1.5 μM.

### Quantitative real-time PCR(qPCR) and reverse transcription PCR (RT-PCR)

TRIzol (Invitrogen, California, USA) was used to extract total RNA from cell lines. 2 μg total RNA was used to synthesis cDNA first-strand by using Reverse Transcriptase Kit (M-MLV) (Promega, Wisconsin, USA). qPCR was performed using the BIO-RAD Real-Time detection system to measure the expression of CHI3L1 in the same cell lines and the details were as follows. Detection of CHI3L1 was performed using FastStart Universal SYBR Green Master (ROX) (Roche, Basel, Switzerland) with PCR cycles set for 10 min at 95 °C, 37 cycles of reaction consisting of 10 seconds at 95 °C, 10 seconds at 60 °C, and 10 seconds at 72 °C. The classic comparative 2^−ΔCt^ method was used to evaluate the relative expression levels of the target genes in the same cell lines.

Two-step RT-PCR was performed to compare the expression of CHI3L1 in different cell lines. First, 2 μg total RNA was used to synthesis cDNA first-strand, then PCR was performed to amplify CHI3L1. Finally, PCR productions of CHI3L1 were running in 1% agarose. After taking photos, quantitative analysis was performed by Image J.

The primer pairs used for amplification of the human CHI3L1 and GAPDH genes were showed in the supplementary.

### Cell migration assay

Cell migration assay was performed using transwell chambers with 8 μm pore size filter membrane (Corning, Shanghai, China). HepG2 and Bel7404 were harvested and suspended with serum-free RPMI 1640 or DMEM, and then seeded into the upper chambers (1 × 10^5^ cells/chambers), with the lower chambers containing 250 μl complete culture medium. After incubation 10 hours at 37 °C and 5% CO_2_ incubator, the inner side of the membranes were cleaned with wet cotton swabs to remove non-migrated cells. The outer sides of the membranes were washed with PBS twice, and then fixed with precooled methanol for 10 minutes and stained with crystal violet. The number of migrated cells for each filter membrane was calculated as the average of 5 random 300 μm × 300 μm fields. All of the experiments were performed three times to obtain robust results.

### Cell invasion assays

Cell invasion assay was also performed with transwell chambers with 8 μm pore size filter membrane. First, the filter membrane is coated with ECM matrigel (BD, New Jersey, USA). Cells (1 × 10^5^) were cultured for 48 hours after seeding into the upper chamber with serum-free medium. The number of invasive cells was calculated as the average of 5 random 300 μm × 300 μm fields of each filter membrane as for the migration assay. All of the experiments were performed three times to obtain robust results.

### Wound-healing cell migration assay

Cells were seeded into six-well plates and grown to confluence. Scratches were made with pipette tips and the wells were washed with PBS twice to remove any cell debris. RPMI 1640 or DMEM containing 1% FBS were added to the wells. The cells were incubated at 37 °C and images were captured at 24 hours after making scratches. The gap distance was evaluated using Image J. All of experiments were repeated three times to obtain robust results.

### *In vivo* tumor growth and metastasis assay

All experimental protocols were approved by the Ethics Committee for Animal Experimentation of Zhejiang University. The BALB/c nude mice (CAnN.Cg-Foxn1^nu^/Crl) used in these experiments were purchased from the animal experimental center of Zhejiang University and the animals were maintained in specific-pathogen-free (SPF) environment. First, cells were counted with blood cell counting chamber, and 5 × 10^6^ cells were injected into the right forelimb armpit and tail vein of mice for *in vivo* tumor growth assessment and metastasis assay respectively. The tumor volume was measured every three days by (L × W × W)/2 (L: tumor length, W: tumor width) method and the mice were sacrificed three weeks or 30 days after cells were injected. For metastasis assay, the lungs of mice were fixed in 4% paraformaldehyde (PFA), washed in PBS. Then dehydrated in ethanol and embedded in paraffin blocks. 5 µm thick tissue sections were prepared and used for H&E staining.

### MTT cell proliferation assay

One thousand cells were plated into 96-wells plates and incubated at 37 °C for 4 days and the medium was changed every other day. MTT (0.5 mg/ml) (Sangon Biotech, Shanghai, China) were added to the well at 8 hours (h), 24 h, 48 h, 72 h and 96 h after cells were plated. DMSO (Sangon Biotech, Shanghai, China) was applied to dissolve formazon crystals after the MTT incubation. At last, OD values at 490 nm were measured for every well. All samples were evaluated in six replicates in three independent experiments.

### Cell cycle analysis with flow cytometry

Cells were cultured with serum-free medium overnight to synchronize the cell cycle of the cells and then cultured with complete medium for 24 h. Cells were harvested and washed with precooled PBS, and fixed with ethanol at 4 °C overnight.

After washing with PBS, cells were resuspended with 500 μl PBS containing PI (50 μg/ml), RNaseA (100 μg/ml), 0.2% TritonX-100 and incubate 30 min at 37 °C by avoiding light. Cells (1 × 10^4^ cells) were analyzed on flow cytometry (ACEA bioscience) and the data were analyzed by the FlowJo program, a software package for analyzing flow cytometry data (www.flowjo.com). Univariate model was used for the analysis of the G0/G1 peak, the S Phase and the G2/M peak excluding a calculation of cell debris and fixation artifacts. Percentages of cells in the G0/G1, the S, and the G2/M phase of cell cycle were determined by three independent experiments.

### Recombinant CHI3L1 production and purification

The CHI3L1 plasmid with 6×-His tag at the C-terminal, and was transfected into HEK-293 T cells to generate stable expression cells. A large number of cells were cultured with the Cell Culture Factory (Corning, Shanghai, China) and supernatant of the cultured cells were collected to purify the protein. The rCHI3L1 (Recombinant CHI3L1) was purified by a commercial company (Suzhou Institute of Tongji University, Jiangsu, China). In brief, Ni-NTA Resin was used to bind the CHI3L1-His tag recombinant protein, and then the protein was eluted with different concentrations of imidazole. SDS-PAGE was performed to detect rCHI3L1.

### Western blot

RIPA (Invitrogen, California, USA) was used to extract the total proteins from cells, and the BCA assay kit (Invitrogen, California, USA) was used to determine the concentration of protein. 25 μg of the total proteins was loaded into the wells of SDS-PAGE along with the molecular weight markers. After running gel for 1 hour, the proteins were transferred onto PVDF membranes, and then the membrane was blocked with 5% skimmed milk or BSA in 1× TBST buffer. Respective primary and secondary antibodies were used to detect the expression of target proteins. Antibodies used in western blot include CHI3L1, GAPDH (Abcam, Shanghai, China), SMAD2, phosphor-SMAD2, SMAD3, phosphor-SMAD3 (Cell signaling, Shanghai, China).

### RNA-seq and bioinformatics analysis

RNA-seq was performed by a commercial company (Genergy Biotechnology, China). For bioinformatics analysis of the RNA-seq data, Trimmomatic^13^, a flexible trimmer for Illumina sequence data, was used to trim the known adaptor sequences from the raw reads with base quality control. The parameters used were: LEADING:20, TRAILING:20, SLIDINGWINDOW:5:20, ILLUMINACLIP: adaptors.fa:2:30:1. The trimmed reads were then mapped against GRCh37 using Tophat^14^ with the parameters (-p 20, -G Ensembl version75 GRCh37, other parameters as defaults). HTseq-count (https://htseq.readthedocs.io/en/release_0.9.1/) was used to assign reads to genes (Ensembl Gene ID). Then DEGseq was used to identify significant differentially expressed genes using default parameters including multiple test corrections^15^. Genes with p-value < 0.001 were considered significantly differential expressed genes.

### Statistical analysis

The data were shown as the mean ± SD. T-test was used for all pair-wise comparisons. A p-value < 0.05 was considered statistically significant.

## Results

### CHI3L1 promotes HCC cell migration and invasion *in vitro* and *in vivo*

**Figure 1:**
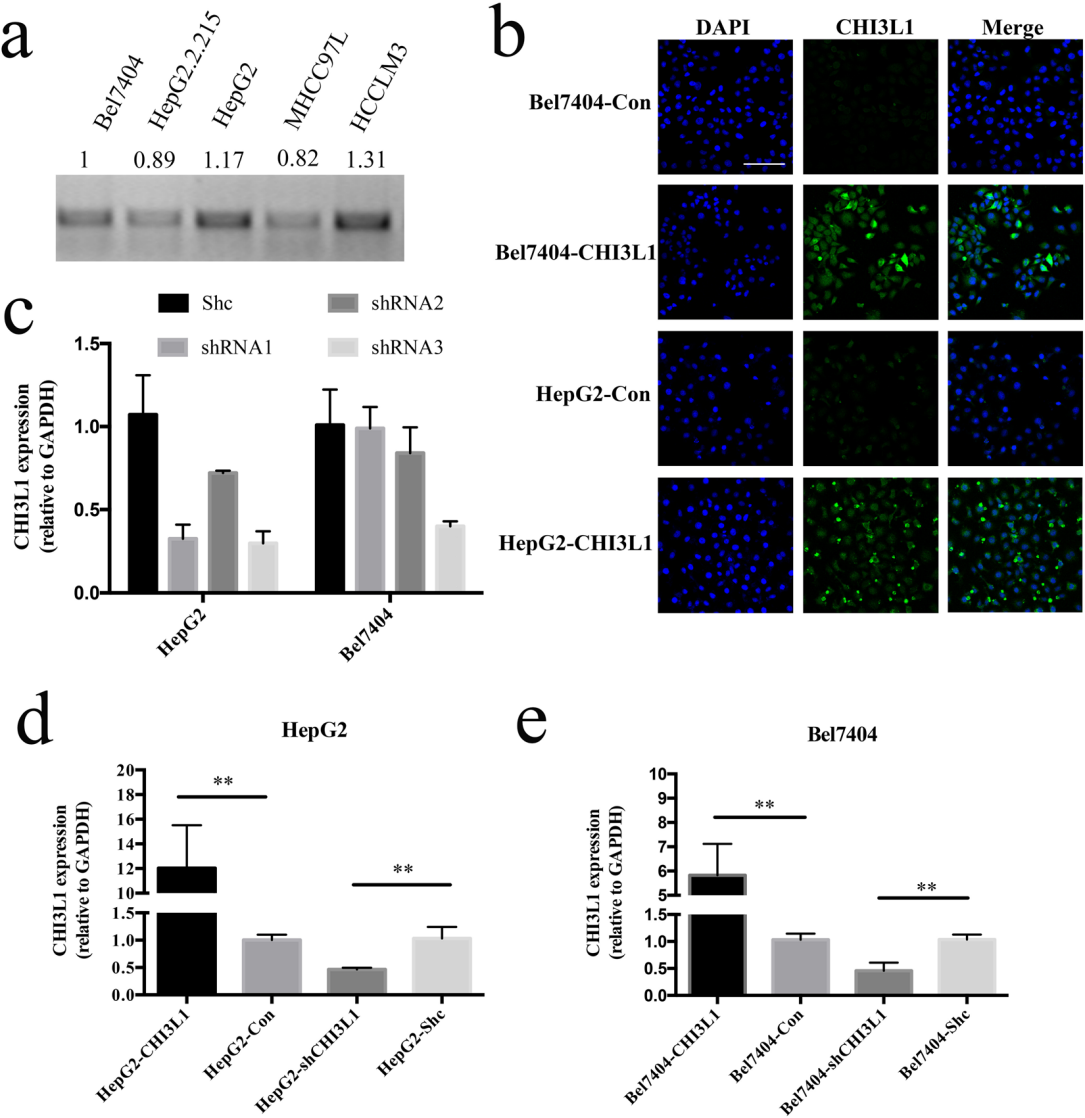
The generation of the stable overexpression and knockdown cell lines. (**a**) The expression of CHI3L1 in the HepG2, HepG2.2.15, Bel7404, MHCC97L and HCCLM3 by Two-step RT-PCR and the grayscale of the strips were showed. (**b**) Immunofluorescence was showed CHI3L1 overexpression in HepG2 and Bel7404 cells. Cell nucleus was stained with DAPI (blue fluorescence) and CHI3L1 was stained with FITC (green fluorescence). (**c**) The expression of CHI3L1 in the HepG2 and Bel7404 cells after transfected with three short-hairpin RNAs. All samples were normalized to the housekeeping gene GAPDH. (**d**) qPCR showing the expression of CHI3L1 in the CHI3L1 overexpression and knockdown cell lines. Scale bar represents 100 μm. Error bars represents SD calculated from three independent experiments. **Represents p < 0.01 by two-tailed t test.

To study the function of CHI3L1, we chose to generate CHI3L1 stable overexpression and knockdown cell lines. In order to decide which cells to use for overexpression and knockdown experiments, we analyzed the expression of CHI3L1 in HepG2, HepG2.2.15, Bel7404, MHCC97L and HCCLM3 by two-step RT-PCR and the grayscale of the strips were showed on the top of strip (Fig. 1a). We chose two cell lines HepG2 and Bel7404 because their expression of CHI3L1 is in the middle range. After the selection of the stable clones, CHI3L1 Immunofluorescence analysis was conducted to confirm the overexpression of CHI3L1 in CHI3L1-overexpression cells Bel7404-CHI3L1 and HepG2-CHI3L1 compared with that in the control cells (Fig. 1b).

To construct CHI3L1 stable knockdown cell lines, quantitative real-time PCR(qPCR) was performed to choose the best short hairpin RNA (shRNA) from three selected putative shRNAs (shRNA1, shRNA2, shRNA3). shRNA3 had the highest knockdown efficiency, which had over 50% suppression in both HepG2 and Bel7404 cells (Fig. 1c) and was therefore used to generate knockdown cell line HepG2-shCHI3L1 and Bel7404-shCHI3L1. qPCR were performed to validate the changes of CHI3L1 protein expression levels in both the overexpression and knockdown stable cell lines (Fig. 1d,e).

**Figure 2:**
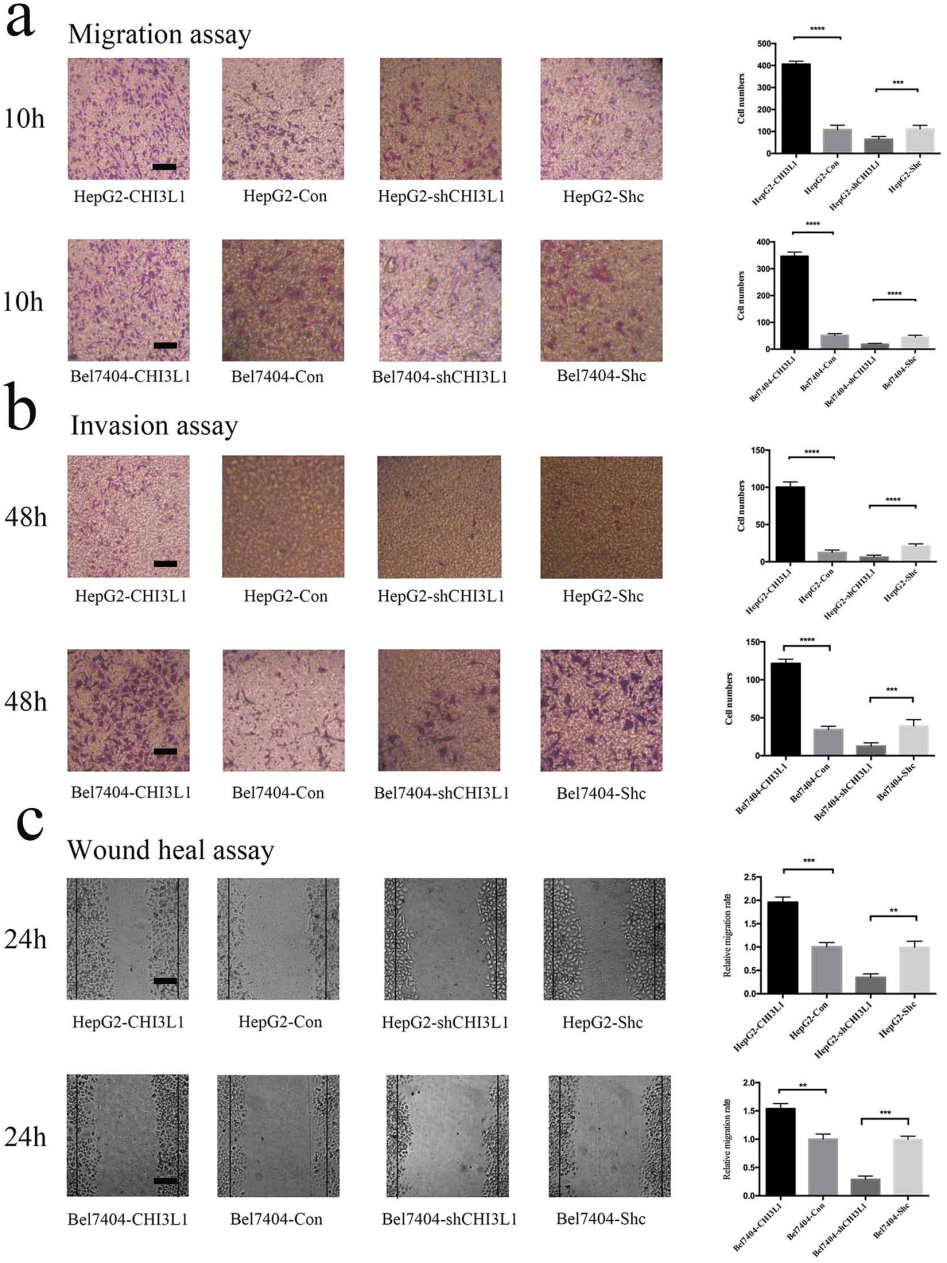
CHI3L1 promotes cancer cell migration and invasion *in vitro*. (**a**) Eight stable transfected cell lines (HepG2-CHI3L1, HepG2-Con, HepG2-shCHI3L1, HepG2-Shc; Bel7404-CHI3L1, Bel7404-Con, Bel7404-shCHI3L1, Bel7404-Shc) were seeded into the upper chambers of transwell plates and complete culture medium was added to the lower chambers. Numbers of migration cells were counted and image were taken at 10 hours after seeding the cells. (**b**) The invasion assays were performed on these 8 stable transduced cell lines. Numbers of invasion cells were counted and image were taken at 48 hours after seeding the cells. (**c**) Cells were seeding into 6-wells plates. The images were taken at 48 hours after the scratches were made. **, *** and **** represent p < 0.05, p < 0.01 and p < 0.001 by two-tailed t test. Scale bar represents 50 μm. Error bar represents SD calculated from three independent experiments.

To study the role of CHI3L1 in migration and invasion of liver cancer cells, we performed transwell migration, invasion and wound healing assays. In the transwell migration assay, we found that the number of migrated cells in HepG2-CHI3L1 was almost three times higher than that in HepG2-Con (p < 0.001) (Fig. 2a). In parallel, the reduction of CHI3L1 in HepG2 (HepG2-shCHI3L1) significantly decreased the number of migrated cells compared with that in the control (HepG2-Shc) (p < 0.001) (Fig. 2a). Similar results were observed for Bel7404 liver cancer cells, suggesting that the effect is probably not cell-type specific (Fig. 2a).

For the transwell invasion assay, we found that the number of invasive cells in HepG2-CHI3L1 was almost four times higher than that of HepG2-Con after an incubation of 48 hours (p < 0.001) (Fig. 2b). Accordingly, the suppression of CHI3L1 in HepG2 significantly decreased the number of invasive cells compared with that in the control HepG2-Shc (p < 0.001) (Fig. 2b). In Bel7404, similar effects were observed but more pronounced as Bel7404-CHI3L1 had more invasive cells than that in the HepG2-CHI3L1 cell (Fig. 2b).

For the wound heal assay, we found that overexpression of CHI3L1 in HepG2 (HepG2-CHI3L1) significantly increased the ability to migrate across the scratch areas (the black line marked the scratch position) (p < 0.001) compared with that in the control cells (HepG2-Con) (Fig. 2c). Accordingly, we found that the knockdown of CHI3L1 in HepG2 (HepG2-shCHI3L1) significantly decreased the ability of cancer cell migration (p < 0.01) (Fig. 2c). Similar trends were found in Bel7404 cells: more Bel7404-CHI3L1 cells migrate to the wound area than those in Bel7404-Con (p < 0.01), and less Bel7404-shCHI3L1 cells to migrate to the wound area than those in Bel7404-Shc (p < 0.001) (Fig. 2c).

**Figure 3:**
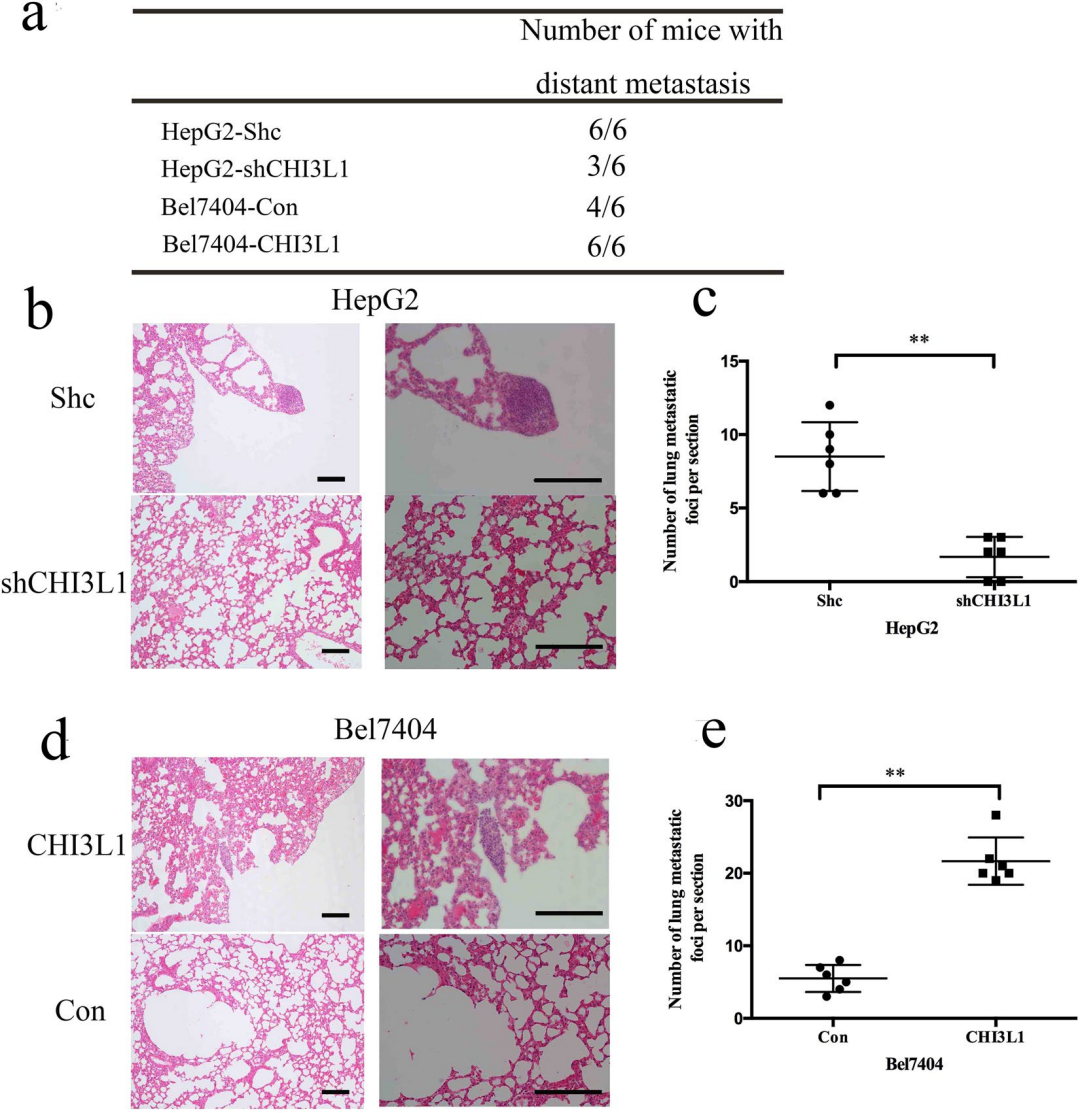
CHI3L1 promotes cancer cell metastasis *in vivo*. (**a**) The total number of mice with distant metastases at 30-days after injection of the HepG2-shCHI3L1, Bel7404-CHI3L1 and the control cells. (**b**,**d**) A representative HE stain picture of the lung of mice injected with the HepG2-Shc, Bel7404-CHI3L1 or their control cells. (**c**,**e**) The numbers of metastatic foci per section in lungs from individual mice injected with the HepG2-shCHI3L1, Bel7404-CHI3L1 and their control cells. Scale bar represent 100 μm (left) or 200 μm (right). ** represents p < 0.01 by two-tailed t test.

To assess the *in vivo* metastatic potential of CHI3L1 overexpression or knockdown in liver cancer cells, we injected the HepG2-shCHI3L1, Bel7404-CHI3L1 and their control cells into the tail veins of nude mice. We found that overexpression of CHI3L1 not only increased the number of mice with lung metastases (Fig. 3a), but also significantly increased the number of lung metastatic foci (Fig. 3d,e). In parallel, when we knockdown the expression of CHI3L1, both of the number of mice with lung metastases and lung metastatic foci in each mouse were decreased significantly (Fig. 3a–c).

### CHI3L1 promotes cell proliferation *in vitro* and *in vivo*

**Figure 4:**
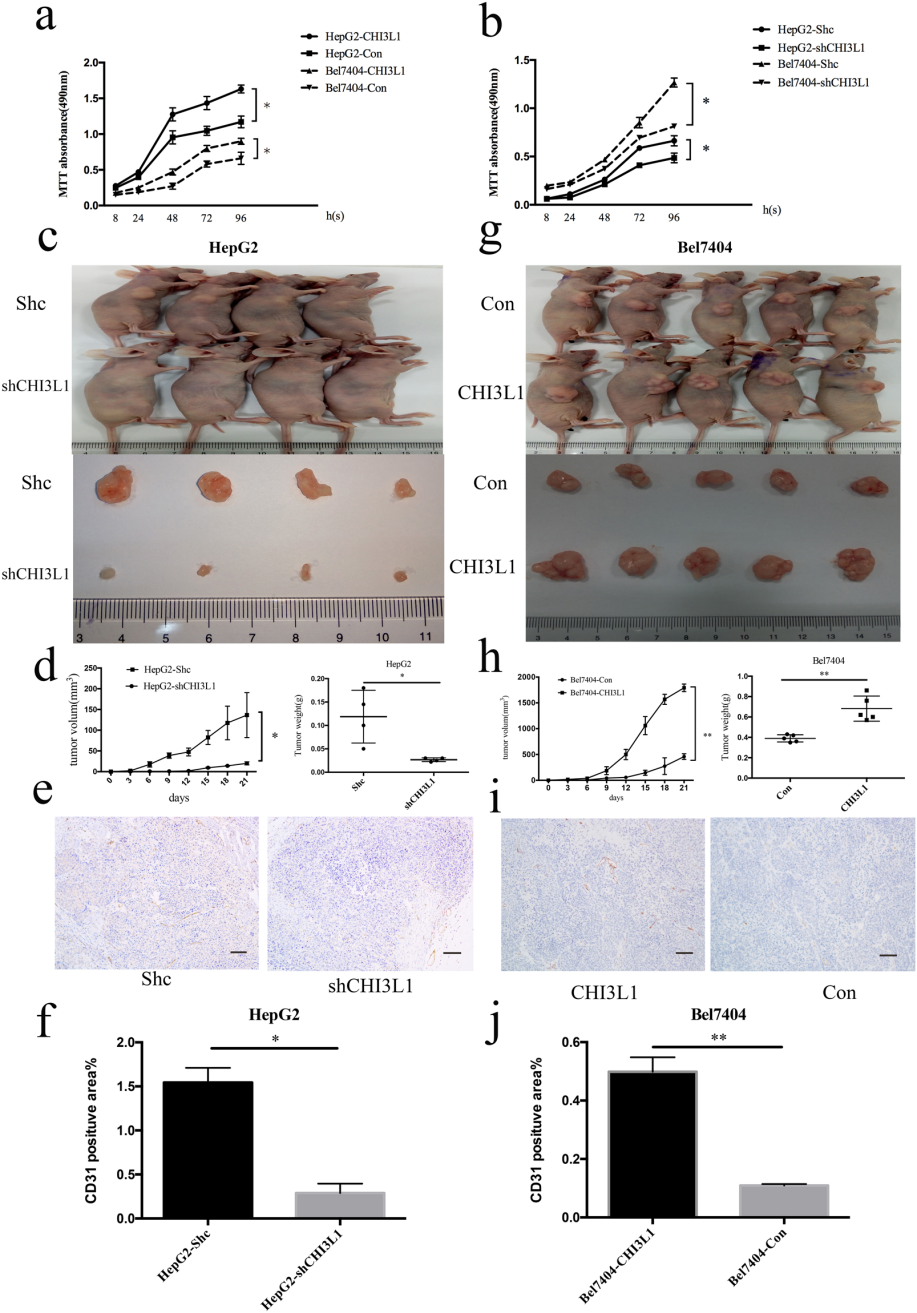
CHI3L1 promotes cancer cell proliferation *in vivo* and *in vitro*. (**a**,**b**) CHI3L1 overexpression and knockdown cells and the control cells were seeded into 96 well plate. Cells were treated with MTT and the MTT absorbance value was measured at 8 h, 24 h, 48 h, 72 h and 96 h after cell seeding. (**c**,**d**,**g**,**h**) Representative images, growth and weight of tumors following subcutaneous injection of the HepG2-shCHI3L1, Bel7404-CHI3L1 or the control cells. (**e**,**i**,**f**,**j**) CD31 stain of the tumors formed by injection of the HepG2-shCHI3L1 and Bel7404-CHI3L1or the control cells. Scale bar represent 200 μm. * and ** represent p < 0.05 and p < 0.01 by two-tailed t test.

We performed an MTT assay for the CHI3L1 overexpression and knockdown in HCC cell lines generated above. We found that the proliferation of CHI3L1 overexpression cell line HepG2-CHI3L1 and Bel7404-CHI3L1 were significantly faster than those in the control cells at 96 hours after seeding the cells (p < 0.05) (Fig. 4a). In parallel, the proliferation of CHI3L1 knockdown cell line HepG2-shCHI3L1 and Bel7404-shCHI3L1 were significantly slower than those in the control cell line HepG2-Shc and Bel7404-Shc at 96 hours after seeding the cells (p < 0.05) (Fig. 4b).

We then injected the HepG2-shCHI3L1, Bel7404-CHI3L1 and their control cells subcutaneously into BALB/c nude mice (CAnN.Cg-Foxn1^nu^/Crl) to investigate the proliferation and growth ability of these cells *in vivo*. Tumor volume was measured every other three day, and the mice were sacrifice three weeks after cell injection (Table 1). The tumor formed by the CHI3L1 knockdown cell (HepG2-shCHI3L1) grew significantly slower than the tumor formed by the control cells (Fig. 4c,d) (p < 0.05). Accordingly, the tumor formed by the CHI3L1 overexpression cell (Bel7404-CHI3L1) grew significantly faster than the tumor formed by the control cells (Fig. 4g,h) (p < 0.01).

**Table 1:** A summary of the sample sizes and tumor weights for the *in vivo* proliferation assay after mice sacrifice.

Sample size (mm^3^)	HepG2-Shc	183	155	150	58	\
	HepG3-shCHI3L1	25	18	17	20	\
Tumor weight (g)	HepG2-Shc	0.18	0.145	0.1	0.05	\
	HepG3-shCHI3L1	0.03	0.022	0.03	0.025	\
Sample size (mm^3^)	Bel7404-Con	368	493	446	486	502
	Bel7404-CHI3L1	1912	1800	1722	1750	1777
Tumor weight (g)	Bel7404-Con	0.36	0.42	0.43	0.35	0.39
	Bel7404-CHI3L1	0.86	0.57	0.6	0.62	0.76

We were curious whether CHI3L1 was also involved in angiogenesis. We therefore analyzed the expression of angiogenesis marker CD31 in the transplanted tumors by immunohistochemistry assay. We found that the expression of CD31 was higher in the tumors formed by the CHI3L1 overexpression cells than the tumors formed by the control cells (Fig. 4i,j). Similarly, we found that the expression of CD31 was lower in the tumors formed by the CHI3L1 knockdown cells than the tumors formed by the control cells (Fig. 4e,f).

**Figure 5:**
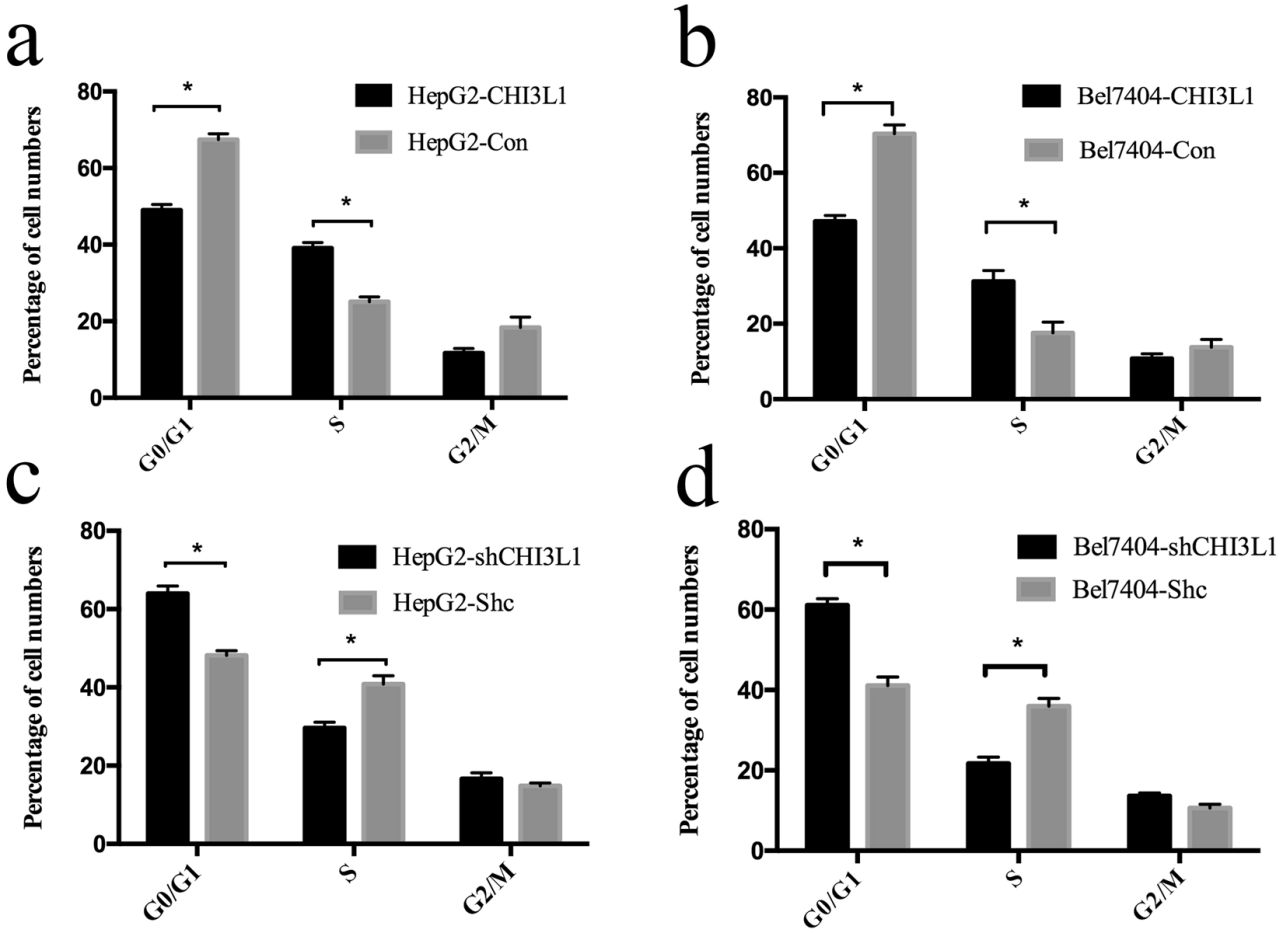
Overexpression of CHI3L1 increased the number of cells in the S stage and decreasing the number of cells in the G0/G1 stages. HepG2 cells with CHI3L1 stable overexpression and knockdown were stained with PI before performing flow cytometry assay. (**a**,**c**) The percentages of cell numbers in different stages were showed as column chart. (**b**,**d**) The same assays were performed on Bel7404 cells with CHI3L1 overexpression and knockdown. The percentages of cell numbers in different stages were showed as column chart. *Means p < 0.05 by two-tailed t test.

In order to see whether changes in cell cycles were associated with changes in cell proliferation by CHI3L1. We used flow cytometry assay to analyze the changes of cell cycles. We found that overexpression of CHI3L1 could significantly increase the number of cells in the S stage and significantly decrease the number of cells in the G0/G1 stage in HepG2 and Bel7404 (p < 0.05) (Fig. 5a,b). By contrast, the reduction of the CHI3L1 expression significantly decreased the number of cells in the S stage and significantly increased the number of cells in the G0/G1 stage in HepG2 and Bel7404 (p < 0.05) (Fig. 5c,d). These results suggested CHI3L1 could promote cancer cell proliferation by regulating the cell cycle of cancer cells.

### Global analysis of the expression profiles of overexpression and knockdown CHI3L1 cells

In order to understand the global molecular mechanism of actions of CHI3L1 in HCC, we used RNA-seq, an accurate digital expression profiling technology based on next generation sequencing, to analyze expression profiles of CHI3L1 overexpression cell lines versus mock-control cell lines in two HCC cell lines (i.e. HepG2-CHI3L1 vs. HepG2-Con; Bel7404-CHI3L1 vs. Bel7404-Con). HTseq-count (https://htseq.readthedocs.io/en/release_0.9.1/) and DEGseq^15^. were used to identify significant differentially expressed genes (DEGs). We identified 43 and 37 genes were significant up- or down-regulated DEGs in both cell lines suggesting a cell-type specific effect of CHI3L1’s action (Supplementary Table S2).

Functional annotation analysis using DAVID (https://david.ncifcrf.gov/) for gene ontology (level 3) revealed that the up-regulated DEGs by CHI3L1 overexpression were significant enriched in cellular component GO:0031982~vesicle, GO:0070062~extracellular exosome, GO:1903561~extracellular vesicle, GO:0043230~ extracellular organelle, GO:0005925~focal adhesion, GO:0005924~cell-substrate adherens junction, GO:0005912~adherens junction (Supplementary Table 3). Amazingly, there were 27 genes (accounting for 79% of the number of genes in the category) involved in GO:0031982~vesicle (Table 2), suggesting that CHI3L1 was actively involved in the production of vesicles. In addition, 11 genes (accounting for 32% of the number of genes in the category) mapped to GO:0005912 (adherens junction) including AHNAK nucleoprotein (AHNAK), LDL receptor related protein 1(LRP1), WAS protein family member 2(WASF2), epidermal growth factor receptor (EGFR), filamin A (FLNA), filamin B (FLNB), heat shock protein family A (Hsp70) member 5 (HSPA5), insulin like growth factor 2 receptor (IGF2R), integrin subunit alpha 5(ITGA5), prolyl 4-hydroxylase subunit beta (P4HB), protein disulfide isomerase family A member 3(PDIA3). In addition, DEGs regulated by CHI3L1 were also enriched in up keywords: phosphoprotein, which suggested CHI3L1 may involve in regulating phosphorylation of protein to active some pathways.

**Table 2:** The list of 27 up-regulated DEGs by CHI3L1 overexpression involved in GO term vesicle (GO:0031982) by cellular component gene ontology.

ENSEMBL_GENE_ID	Gene Name
ENSG00000185567	AHNAK nucleoprotein 2(AHNAK2)
ENSG00000124942	AHNAK nucleoprotein (AHNAK)
ENSG00000123384	LDL receptor related protein 1(LRP1)
ENSG00000158195	WAS protein family member 2(WASF2)
ENSG00000090861	alanyl-tRNA synthetase (AARS)
ENSG00000126001	centrosomal protein 250(CEP250)
ENSG00000133048	chitinase 3 like 1(CHI3L1)
ENSG00000197102	dynein cytoplasmic 1 heavy chain 1 (DYNC1H1)
ENSG00000146648	epidermal growth factor receptor (EGFR)
ENSG00000196924	filamin A (FLNA)
ENSG00000136068	filamin B (FLNB)
ENSG00000105220	glucose-6-phosphate isomerase (GPI)
ENSG00000089597	glucosidase II alpha subunit (GANAB)
ENSG00000044574	heat shock protein family A (Hsp70) member 5(HSPA5)
ENSG00000180573	histone cluster 1 H2A family member c (HIST1H2AC)
ENSG00000197081	insulin like growth factor 2 receptor (IGF2R)
ENSG00000146674	insulin like growth factor binding protein 3(IGFBP3)
ENSG00000161638	integrin subunit alpha 5(ITGA5)
ENSG00000183853	kin of IRRE like (Drosophila) (KIRREL)
ENSG00000102144	phosphoglycerate kinase 1(PGK1)
ENSG00000183255	pituitary tumor-transforming 1 interacting protein (PTTG1IP)
ENSG00000185624	prolyl 4-hydroxylase subunit beta (P4HB)
ENSG00000167004	protein disulfide isomerase family A member 3(PDIA3)
ENSG00000142949	protein tyrosine phosphatase, receptor type F (PTPRF)
ENSG00000116260	quiescin sulfhydryl oxidase 1(QSOX1)
ENSG00000198431	thioredoxin reductase 1(TXNRD1)
ENSG00000152291	trans-golgi network protein 2(TGOLN2)

Interestingly, we noted that the up-stream sequence feature–short sequence motif: Prevents secretion from ER–was significantly enriched (Benjamini adjusted P value of 6.0E-3). The genes contain this short sequence motif are: heat shock protein family A (Hsp70) member 5 (HSPA5), prolyl 4-hydroxylase subunit beta (P4HB), protein disulfide isomerase family A member 3 (PDIA3). Up-regulation of these genes by CHI3L1 potentially prevents proteins secretion from ER.

For the both down-regulated genes, GO biological process GO:0022900 electron transport chain or GO cellular component terms GO:0070469~respiratory chain with 9 genes (Supplementary Table 4): MTND2 (ND2), NADH dehydrogenase, subunit 4 (complex I) (ND4), NADH dehydrogenase, subunit 6 (complex I) (ND6), cytochrome b (CYTB), cytochrome c oxidase III (COX3), cytochrome c oxidase subunit 7B(COX7B), cytochrome c oxidase subunit I(COX1), cytochrome c oxidase subunit II (COX2), ubiquinol-cytochrome c reductase binding protein (UQCRB), suggesting that the down-regulated genes are mostly related to mitochondrial functions.

### CHI3L1 activates the TGF-β pathways

**Figure 6:**
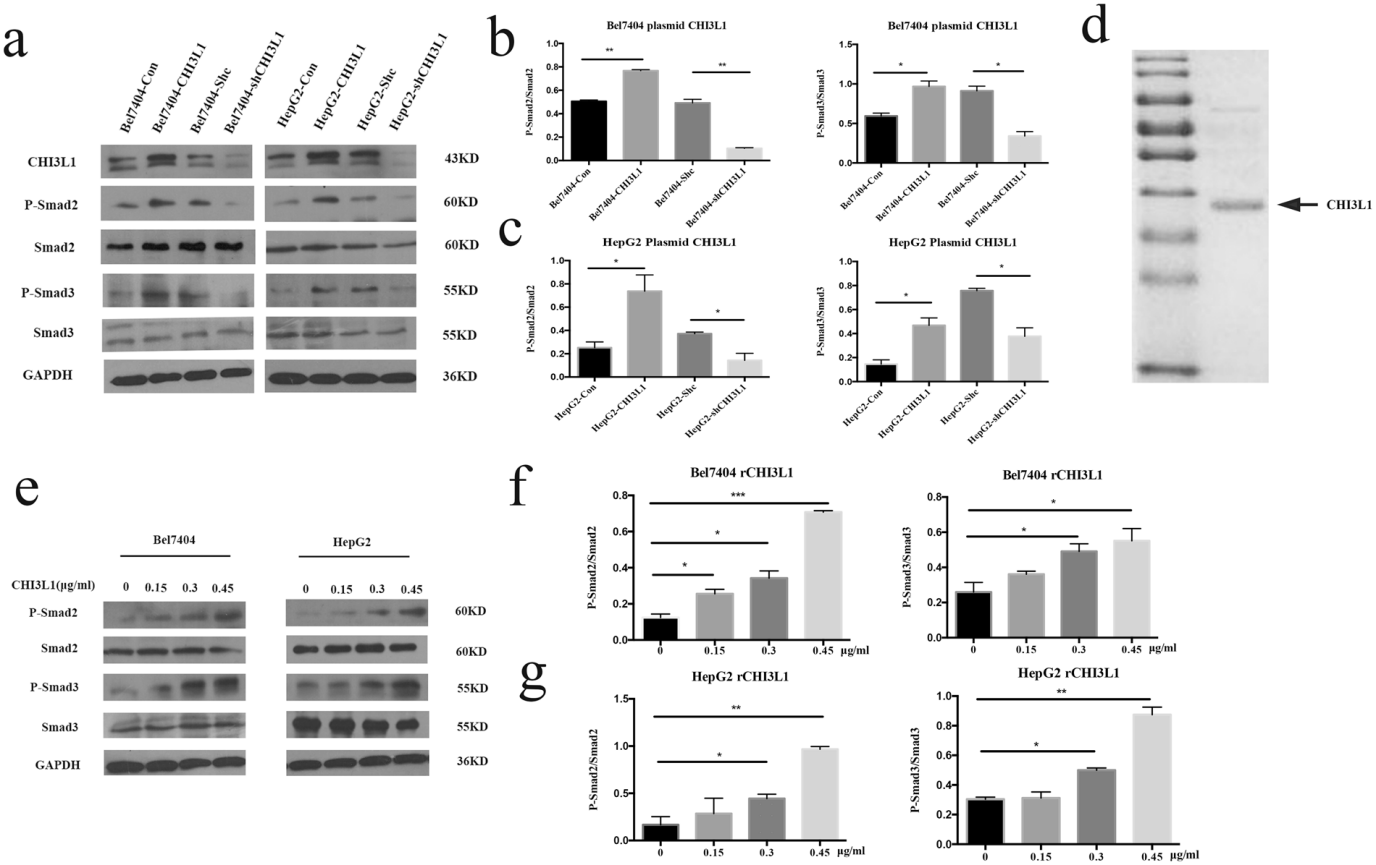
CHI3L1 can activate TGF-β signaling pathway. (**a**–**c**) Western blot showing CHI3L1 activated the TGF-β signaling pathway in Bel7404 and HepG2 cells. (**d**) The SDS-PAGE picture of rCHI3L1. The arrow points to the protein band representing rCHI3L1. (**e**–**g**) TGF-β signaling pathway could be activated by rCHI3L1 in Bel7404 and HepG2 cells.

We next investigated the changes of protein expression or phosphorylation using hypothesis driven approach instead of global approach. First, we evaluated the CHI3L1 expression in all stable cell lines (Fig. 6a). As CHI3L1 was involved in liver fibrosis and liver fibrosis was regulated by TGF-β and AKT pathway^16^, we therefore used Western blot assay to investigate the relationship between CHI3L1 and TGF-β pathway. We found the overexpression of CHI3L1 upregulated P-SMAD2, P-SMAD3 and the suppression of CHI3L1 downregulated the P-SMAD2, P-SMAD3 levels in both Bel7404 and HepG2 cells (Fig. 6a–c).

We further proved the effect of CHI3L1 by adding *in vitro* purified recombinant CHI3L1 (rCHI3L1) (Fig. 6d) at different concentration (0, 0.15, 0.3, 0.45 µg/ml) to the cell culture medium for a short period of 3 hours incubation. We found the P-Smad2 and P-Smad3 increased with increasing concentration of rCHI3L1 in culture medium (Fig. 6e–g). Our data for the first time suggests CHI3L1 could active TGF-β by upregulating the phosphorylation of SMAD2 and SMAD3.

## Discussion

In our study, we found that the overexpression of CHI3L1 can promote cell proliferation, migration and invasion using *in vitro* in HepG2 and Bel7404 liver cancer cell model (Figs 1–2) and *in vivo* using mice models (Figs 3–4). We further showed that overexpression of CHI3L1 significantly increased the number of cells in S stage and decreased the number of cells in G0/G1 stage in both HepG2 and Bel7404, suggesting that CHI3L1 promoted cancer cell proliferation by regulating cell cycles. Our data are consistent with previous finding that higher expression of CHI3L1 in sera of several types of cancers including breast, ovarian, colon and lung. In HCC, Pan *et al*. find the mRNA and protein levels of CHI3L1 in HCC tissues were up-regulated compared with those in adjacent peritumoral tissues and further increased in metastatic tumor (P < 0.05) in 19 pairs of HCC specimens, and they further show that HCC patients with positive CHI3L1 expression have worse overall survival and disease-free survival compared with those with negative CHI3L1 expression (P < 0.001, respectively) by Kaplan-Meier survival analysis. Our previous studies also found the expression of CHI3L1 was higher in liver cancer tissues than adjacent normal tissues by proteomics and transcritomics^11^. However, these previous studies do not analyze the underlying mechanism of the CHI3L1 in HCC. Here we showed that CHI3L1 promotes cancer cell proliferation by increasing the number of cells in the S stage (Fig. 5).

To gain a more comprehensive understanding of the mechanism of the CHI3L1’s global impact on liver cancer cells, we did RNA-seq analysis to gain a more comprehensive genome-wide understanding of the gene expression changes. We found that CHI3L1 was a key player in extracellular spaces (exosome and vesicles) and in adherens junction (Table 1). This data is consistent with the current understanding of the functions of CHI3L1 in liver fibrosis, which is hallmarked by excess accumulation of extracellular matrix proteins^17^.

We also found that CHI3L1 regulated genes involved in the process of cell migration (GO:0016477). Previous study has reported that CHI3L1 secreted by colorectal cancer cells could promote macrophage recruitment and increase the density of macrophage in tumor microenvironment^18^ and CHI3L1 expression in the tumor microenvironment promotes cancer metastasis by activating the MAPK and AKT signaling pathway^19^. Our findings suggested that CHI3L1 is probably involved in the regulation of macrophage migration and cell immunity in the HCC.

We also found that several genes up-regulated by CHI3L1 contain the short sequence motif: Prevents secretion from endoplasm reticulum (ER) with a significantly enriched with a Benjamini adjusted P value of 7.1E-2. The genes contain this short sequence motif are: calreticulin (CALR), heat shock protein family A (Hsp70) member 5(HSPA5), prolyl 4-hydroxylase subunit beta (P4HB), protein disulfide isomerase family A member 3(PDIA3). Up-regulation of these genes by CHI3L1 potentially prevents proteins secretion from ER, suggesting that CHI3L1 not only actively participated in extracellular matrix (ECM), but also participated in the regulation of secretion of proteins to the ECM via ER passage.

Function annotation analysis on up-regulated DEGs by CHI3L1 overexpression also enriched in up keywords: phosphoprotein (p-value: 4.31E-07), which was consistent with our following findings CHI3L1 could active TGF-β pathway to up-regulate the phosphorylation of Smad2 and Smad3 by both plasmid transfection and protein adding ways. As we know, interleukin-13 receptor subunit α2 (IL-13Rα2) is the receptor for CHI3L1 and CHI3L1 binds specially to the extracellular domain of IL-13Rα2 to activate PI3K/AKT and MAPK/ERK pathways^20^. In addition, syndecan, αvβ3 and αvβ5 were also reported as ligand for CHI3L1 to activate PI3K/AKT and MAPK/ERK pathways in different cells^21,22^. Fichtner *et al*. reported that IL-13Rα2 is involved in the induction of TGF-β1 production and thereby acted on the TGF-β signaling pathway^23^. In our present study, we showed that CHI3L1 could activate TGF-β signaling pathways by western blot in both vector transfecting and protein stimulating ways. Here, we proposed a hypothesis that CHI3L1 may bind to IL-13Rα2 to produce TGF-β and overexpression of TGF-β could active TGF-β signaling pathway.

In summary, our data added new understanding of the mechanism of CHI3L1’s action. 1) CHI3L1 promoted cancer cell proliferation by regulating cell cycles; 2) CHI3L1 promoted cancer cell invasion and metastasis by acting on the EMT pathways; 3) CHI3L1 regulate liver fibrosis potentially by regulating the TGF-β signaling pathways; 4) CHI3L1 may has direct kinase activities or activate a kinase to phosphorylate SMAD2 and SMAD3.

## Electronic supplementary material

Supplementary Tables and Figures
