# Supplementary material for: CHI3L1 promotes tumor progression by activating TGF-β signaling pathway in hepatocellular carcinoma
Source: Sci Rep. 2018 Oct 9;8:15029. doi: 10.1038/s41598-018-33239-8 (PMC6177412; doi:10.1038/s41598-018-33239-8)
Supplement: Supplementary file 1 — Supplementary Tables and Figures [file 41598_2018_33239_MOESM1_ESM.docx]

**CHI3L1 promotes tumor progression by activating TGF-β signaling pathway in hepatocellular carcinoma**

Qing-Chong Qiu^1,2,3^ , Lin Wang^1,3^, Shan-Shan Jin^1^, Guan-Feng Liu^1^, Jie Liu^1^, Liang Ma^3^, Rui-Fang Mao^3^, Ying-Ying Ma^3^, Na Zhao^3^, Ming Chen^1,3*^, Biao-Yang Lin^1,2,3,4*^

^1^ College of life science, Zhejiang University, Hangzhou, P.R. China

^2^ Collaborative Innovation Center for Diagnosis and Treatment of Infectious Diseases, The First Affiliated Hospital, School of Medicine, Zhejiang University, Hangzhou, China

^3^ Systems Biology Division, Zhejiang-California International Nanosystems Institute (ZCNI), Zhejiang University, Hangzhou, Zhejiang Province, P.R. China

^4^ Department of Urology, University of Washington, Seattle, USA

**Correspondence to**: Biaoyang Lin, email: [Biaoylin@gmail.com](mailto:Biaoylin@gmail.com)

Ming Chen, email: [mchen@zju.edu.cn](mailto:mchen@zju.edu.cn)

Supplementary Tables and Figures

Supplementary Table S1: Sequences of primers used in the paper

Supplementary Table S2: Significantly changed in both HepG2 and Bel7404 cells comparing CHI3L1 overexpressing cell to mock-control cells.

Supplementary Table S3: Functional annotation of the up-regulated DEGs by CHI3L1.

Supplementary Table S4: Functional annotation of the down-regulated DEGs by CHI3L1.

Supplementary Fig S1: Uncropped western blot used in Fig. 1a

Supplementary Fig S1: Uncropped western blot used in Fig. 6a

Supplementary Fig S1: Uncropped western blot used in Fig. 6e

Supplementary Table S1. Sequences of the primers designed for the manuscript.

| **Gene name** | **Sequences 5'-3'** |
| --- | --- |
| **CHI3L1-shRNA1-F** | **GATCCGACTCTCTTGTCTGTCGGATTCAAGAGATCCGACAGACAAGAGAGTCTTA** |
| **CHI3L1-shRNA1-R** | **AGCTTAAGACTCTCTTGTCTGTCGGATCTCTTGAATCCGACAGACAAGAGAGTCG** |
| **CHI3L1-shRNA2-F** | **GATCCGTAAGACTCGGGATTAGTACATTCAAGAGATGTACTAATCCCGAGTCTTACATA** |
| **CHI3L1-shRNA2-R** | **AGCTTATGTAAGACTCGGGATTAGTACATCTCTTGAATGTACTAATCCCGAGTCTTACG** |
| **CHI3L1-shRNA3-F** | **GATCCGGTGCAGTACCTGAAGGATTTCAAGAGAATCCTTCAGGTACTGCACCTTA** |
| **CHI3L1-shRNA3-R** | **AGCTTAAGGTGCAGTACCTGAAGGATTCTCTTGAAATCCTTCAGGTACTGCACCG** |
| **CHI3L1-nonsense shRNA-F** | **GATCCAATTCTCCGAACGTGTCACGTTTCAAGAGAACGTGACACGTTCGGAGAATTA** |
| **CHI3L1-nonsense shRNA-R** | **AGCTTAATTCTCCGAACGTGTCACGTTCTCTTGAAACGTGACACGTTCGGAGAATTG** |
| **CHI3L1 F** | **CCCAACCTGAAGACTCTCTTG** |
| **CHI3L1 R** | **CCAAGATAGCCTCCAACACC** |
| **GAPDH-F** | **TCGACAGTCAGCCGCATCT** |
| **GAPDH-R** | **CCGTTGACTCCGACCTTCA** |

Supplementary Table S2. Significantly changed genes in both HepG2 and Bel7404 cells comparing CHI3L1 overexpressing cell to mock-control cells by RNA-seq analysis.

|  |  | HepG2 | | Bel7404 | |
| --- | --- | --- | --- | --- | --- |
| GeneNames | Gene Symbol | log2(Fold_change) normalized | p-value | log2(Fold_change) normalized | p-value |
| ENSG00000133048 | CHI3L1 | 12.33556159 | 0 | 13.93455873 | 0 |
| ENSG00000123384 | LRP1 | 0.42575185 | 8.29E-125 | 0.064574699 | 5.41E-05 |
| ENSG00000167548 | KMT2D | 0.408332134 | 7.13E-20 | 0.104278967 | 6.39E-04 |
| ENSG00000085982 | USP40 | 0.36367816 | 1.03E-04 | 0.360269219 | 1.77E-04 |
| ENSG00000124942 | AHNAK | 0.284838208 | 5.34E-119 | 0.148236495 | 1.18E-21 |
| ENSG00000146674 | IGFBP3 | 0.273862269 | 2.43E-261 | 0.354189911 | 1.87E-57 |
| ENSG00000197102 | DYNC1H1 | 0.268678273 | 7.92E-67 | 0.055136139 | 4.07E-04 |
| ENSG00000180573 | Hist1h2ac | 0.235552157 | 7.53E-04 | 0.618479141 | 1.99E-06 |
| ENSG00000185567 | AHNAK2 | 0.228347176 | 1.21E-14 | 0.209318867 | 7.94E-22 |
| ENSG00000146648 | EGFR | 0.212872714 | 4.70E-83 | 0.122200204 | 3.45E-10 |
| ENSG00000152291 | TGOLN2 | 0.198473955 | 3.05E-10 | 0.096989955 | 1.69E-04 |
| ENSG00000127483 | HP1BP3 | 0.195099376 | 5.49E-10 | 0.174143797 | 1.47E-06 |
| ENSG00000126001 | CEP250 | 0.185500567 | 6.01E-05 | 0.124664495 | 7.74E-04 |
| ENSG00000136068 | FLNB | 0.183876008 | 5.32E-48 | 0.175654381 | 3.96E-17 |
| ENSG00000167978 | SRRM2 | 0.174736259 | 1.58E-23 | 0.14842301 | 3.23E-13 |
| ENSG00000137076 | MIR6852 | 0.167771469 | 2.24E-16 | 0.116670816 | 2.25E-04 |
| ENSG00000183853 | KIRREL | 0.166067131 | 1.00E-04 | 0.136217705 | 7.02E-04 |
| ENSG00000130702 | MIR4758 | 0.161713413 | 1.04E-14 | 0.110427005 | 4.52E-10 |
| ENSG00000167004 | PDIA3 | 0.154309998 | 1.34E-21 | 0.068722415 | 5.14E-04 |
| ENSG00000116285 | ERRFI1 | 0.151439181 | 2.13E-04 | 0.200502711 | 5.28E-08 |
| ENSG00000062716 | MIR21 | 0.15027786 | 5.02E-04 | 0.155019498 | 3.95E-07 |
| ENSG00000197081 | IGF2R | 0.142321603 | 2.65E-07 | 0.115004763 | 8.21E-06 |
| ENSG00000130723 | PRRC2B | 0.135446206 | 8.58E-08 | 0.142642264 | 4.10E-13 |
| ENSG00000183255 | PTTG1IP | 0.131270576 | 4.35E-12 | 0.139266207 | 6.93E-08 |
| ENSG00000100201 | DDX17 | 0.127822996 | 4.48E-09 | 0.089709729 | 4.95E-04 |
| ENSG00000082641 | NFE2L1 | 0.12658512 | 2.84E-11 | 0.158611196 | 1.41E-07 |
| ENSG00000122884 | P4HA1 | 0.12574093 | 5.84E-06 | 0.18273212 | 1.90E-07 |
| ENSG00000044574 | HSPA5 | 0.122607865 | 3.07E-12 | 0.376947073 | 2.58E-171 |
| ENSG00000114270 | MIR711 | 0.12090325 | 1.66E-04 | 0.078251686 | 5.51E-05 |
| ENSG00000142949 | PTPRF | 0.119842484 | 2.02E-09 | 0.144283748 | 9.35E-13 |
| ENSG00000161638 | ITGA5 | 0.109484076 | 3.95E-08 | 0.213335746 | 2.85E-17 |
| ENSG00000158195 | WASF2 | 0.106323348 | 1.51E-07 | 0.120190854 | 1.60E-07 |
| ENSG00000134013 | LOXL2 | 0.104967084 | 8.94E-17 | 0.126379564 | 2.34E-06 |
| ENSG00000173757 | STAT5B | 0.098480721 | 6.86E-05 | 0.14216406 | 8.34E-07 |
| ENSG00000090861 | AARS | 0.096088767 | 3.08E-06 | 0.181144524 | 1.02E-09 |
| ENSG00000185624 | P4HB | 0.088466233 | 7.95E-15 | 0.067726918 | 1.72E-05 |
| ENSG00000116260 | qsox1 | 0.087284449 | 1.45E-06 | 0.236538285 | 1.86E-10 |
| ENSG00000105220 | GPI | 0.081985968 | 1.64E-16 | 0.077343216 | 7.18E-05 |
| ENSG00000102144 | PGK1 | 0.078300596 | 2.10E-50 | 0.170672982 | 1.66E-67 |
| ENSG00000196924 | FLNA | 0.07036357 | 8.88E-21 | 0.103817924 | 6.15E-32 |
| ENSG00000010292 | NCAPD2 | 0.069729263 | 1.45E-04 | 0.0717888 | 3.91E-04 |
| ENSG00000198431 | TXNRD1 | 0.066008441 | 1.36E-07 | 0.091452941 | 8.06E-09 |
| ENSG00000089597 | GANAB | 0.065620797 | 6.75E-07 | 0.124221005 | 1.49E-09 |
| ENSG00000101160 | CTSZ | -0.073760708 | 1.08E-04 | -0.10215972 | 3.06E-09 |
| ENSG00000130600 | MIR675 | -0.083532349 | 4.48E-99 | -0.171069557 | 1.87E-45 |
| ENSG00000180879 | SSR4 | -0.083648433 | 7.77E-04 | -0.097846801 | 1.85E-04 |
| ENSG00000198804 | COX1 | -0.087438235 | 1.11E-19 | -0.316343058 | 0.00E+00 |
| ENSG00000198938 | COX3 | -0.087876485 | 4.04E-06 | -0.438572487 | 0.00E+00 |
| ENSG00000112473 | SLC39A7 | -0.093457247 | 6.46E-05 | -0.101112792 | 1.38E-04 |
| ENSG00000198727 | CYTB | -0.094238515 | 3.37E-04 | -0.424246647 | 9.35E-185 |
| ENSG00000115053 | NCL | -0.097464903 | 1.32E-07 | -0.072487196 | 7.78E-06 |
| ENSG00000122566 | HNRNPA2B1 | -0.102060058 | 4.93E-22 | -0.095862393 | 1.44E-18 |
| ENSG00000102265 | TIMP1 | -0.103118032 | 7.38E-05 | -0.122554122 | 5.31E-07 |
| ENSG00000077942 | FBLN1 | -0.111251946 | 1.63E-13 | -0.073071457 | 1.41E-04 |
| ENSG00000080824 | HSP90AA1 | -0.111870023 | 7.54E-17 | -0.260795267 | 1.22E-118 |
| ENSG00000156467 | Uqcrb | -0.113117624 | 4.75E-04 | -0.181786332 | 6.09E-07 |
| ENSG00000131828 | PDHA1 | -0.118778156 | 2.97E-07 | -0.080934495 | 7.13E-04 |
| ENSG00000169908 | TM4SF1 | -0.125184054 | 1.12E-19 | -0.19470522 | 3.24E-31 |
| ENSG00000198763 | ND2 | -0.13098744 | 7.29E-07 | -0.451943725 | 1.20E-158 |
| ENSG00000198899 | ATP6 | -0.135250356 | 2.04E-06 | -0.565888952 | 0.00E+00 |
| ENSG00000198242 | RPL23A | -0.137399317 | 7.45E-06 | -0.202768601 | 8.81E-20 |
| ENSG00000131462 | TUBG1 | -0.144705859 | 4.26E-04 | -0.233979103 | 3.99E-08 |
| ENSG00000198886 | ND4 | -0.144847334 | 1.03E-50 | -0.445972052 | 0.00E+00 |
| ENSG00000128708 | HAT1 | -0.150450143 | 6.42E-04 | -0.306731266 | 1.30E-07 |
| ENSG00000198695 | ND6 | -0.155488982 | 3.24E-08 | -0.596901619 | 9.30E-129 |
| ENSG00000124207 | CSE1L | -0.156979371 | 5.66E-05 | -0.22193917 | 7.07E-21 |
| ENSG00000132906 | CASP9 | -0.158872318 | 4.95E-05 | -0.276274334 | 2.87E-04 |
| ENSG00000198712 | COX2 | -0.159135946 | 1.23E-38 | -0.304320818 | 0.00E+00 |
| ENSG00000169710 | FASN | -0.162521022 | 1.45E-14 | -0.096533935 | 3.19E-05 |
| ENSG00000120694 | HSPH1 | -0.178698868 | 5.17E-04 | -0.363559493 | 1.35E-18 |
| ENSG00000141232 | TOB1 | -0.186393911 | 4.82E-06 | -0.196958911 | 9.81E-05 |
| ENSG00000131174 | COX7B | -0.187251065 | 6.00E-10 | -0.123487822 | 4.74E-05 |
| ENSG00000123358 | NR4A1 | -0.190294942 | 4.49E-04 | -0.230791432 | 5.00E-05 |
| ENSG00000127129 | EDN2 | -0.217600551 | 1.20E-05 | -0.455658009 | 9.00E-04 |
| ENSG00000065548 | ZC3H15 | -0.218738417 | 1.88E-04 | -0.189236328 | 3.52E-04 |
| ENSG00000142089 | IFITM3 | -0.241784637 | 5.87E-17 | -0.131673254 | 3.87E-06 |
| ENSG00000160131 | VMA21 | -0.250509268 | 8.00E-04 | -0.192630915 | 2.86E-04 |
| ENSG00000185201 | IFITM2 | -0.304772698 | 7.14E-13 | -0.25357683 | 1.37E-08 |
| ENSG00000053108 | FSTL4 | -0.418616701 | 2.75E-05 | -0.139404578 | 2.19E-06 |
| ENSG00000268205 | GC19P057817, GC19P057818 | -1.198477069 | 5.42E-10 | -1.072160742 | 4.98E-06 |

Supplementary Table S3. Functional annotation of the up-regulated DEGs by CHI3L1.

| Category | Term | PValue | FDR |
| --- | --- | --- | --- |
| GOTERM_CC_3 | GO:0031982~vesicle | 2.29E-08 | 1.92E-07 |
| UP_KEYWORDS | Phosphoprotein | 4.31E-07 | 3.79E-06 |
| GOTERM_CC_DIRECT | GO:0070062~extracellular exosome | 1.09E-06 | 1.01E-05 |
| GOTERM_CC_3 | GO:0070062~extracellular exosome | 8.72E-07 | 1.46E-05 |
| GOTERM_CC_3 | GO:1903561~extracellular vesicle | 6.36E-07 | 1.60E-05 |
| GOTERM_CC_3 | GO:0043230~extracellular organelle | 4.80E-07 | 1.61E-05 |
| GOTERM_CC_DIRECT | GO:0005925~focal adhesion | 4.05E-06 | 7.44E-05 |
| GOTERM_CC_3 | GO:0005924~cell-substrate adherens junction | 2.28E-06 | 9.55E-05 |
| GOTERM_CC_3 | GO:0005912~adherens junction | 2.31E-05 | 1.16E-03 |
| UP_KEYWORDS | Acetylation | 6.87E-04 | 1.21E-02 |
| GOTERM_CC_DIRECT | GO:0031012~extracellular matrix | 1.11E-03 | 3.08E-02 |
| UP_KEYWORDS | Ubl conjugation | 1.57E-03 | 4.14E-02 |

Supplementary Table S4. Functional annotation of the down-regulated DEGs by CHI3L1.

| Category | Term | P-Value | FDR |
| --- | --- | --- | --- |
| GOTERM_MF_DIRECT | GO:0004129~cytochrome-c oxidase activity | 1.99E-05 | 0.022673824 |
| GOTERM_CC_DIRECT | GO:0005739~mitochondrion | 1.80E-05 | 0.019807616 |
| GOTERM_CC_DIRECT | GO:0005743~mitochondrial inner membrane | 8.04E-08 | 8.84E-05 |
| GOTERM_CC_3 | GO:0005746~mitochondrial respiratory chain | 6.41E-07 | 7.41E-04 |
| GOTERM_BP_3 | GO:0006091~generation of precursor metabolites and energy | 2.62E-08 | 3.57E-05 |
| GOTERM_BP_DIRECT | GO:0006123~mitochondrial electron transport, cytochrome c to oxygen | 7.01E-06 | 0.009146299 |
| GOTERM_MF_3 | GO:0015002~heme-copper terminal oxidase activity | 2.52E-05 | 0.025028962 |
| GOTERM_BP_3 | GO:0015980~energy derivation by oxidation of organic compounds | 1.41E-09 | 1.92E-06 |
| GOTERM_MF_3 | GO:0016675~oxidoreductase activity, acting on a heme group of donors | 2.78E-05 | 0.027674457 |
| GOTERM_BP_3 | GO:0019637~organophosphate metabolic process | 2.71E-05 | 0.036974526 |
| GOTERM_CC_3 | GO:0019866~organelle inner membrane | 6.79E-07 | 7.86E-04 |
| GOTERM_BP_3 | GO:0022900~electron transport chain | 2.76E-11 | 3.77E-08 |
| GOTERM_CC_3 | GO:0031966~mitochondrial membrane | 4.03E-06 | 0.004658352 |
| GOTERM_CC_3 | GO:0031967~organelle envelope | 5.34E-06 | 0.006178922 |
| GOTERM_CC_3 | GO:0031975~envelope | 5.57E-06 | 0.006442474 |
| GOTERM_CC_3 | GO:0044429~mitochondrial part | 1.01E-05 | 0.011629785 |
| GOTERM_CC_3 | GO:0044455~mitochondrial membrane part | 2.00E-06 | 0.002310773 |
| GOTERM_CC_3 | GO:0045277~respiratory chain complex IV | 4.20E-06 | 0.004855668 |
| GOTERM_BP_3 | GO:0055086~nucleobase-containing small molecule metabolic process | 6.41E-07 | 8.73E-04 |
| GOTERM_BP_3 | GO:0055114~oxidation-reduction process | 9.55E-06 | 0.013005916 |
| GOTERM_CC_3 | GO:0070069~cytochrome complex | 4.18E-09 | 4.84E-06 |
| GOTERM_CC_3 | GO:0070469~respiratory chain | 8.33E-12 | 9.63E-09 |
| GOTERM_CC_3 | GO:0098796~membrane protein complex | 2.05E-05 | 0.023677261 |
| GOTERM_CC_3 | GO:0098798~mitochondrial protein complex | 1.54E-05 | 0.017833985 |
| GOTERM_CC_3 | GO:0098800~inner mitochondrial membrane protein complex | 4.95E-06 | 0.005723165 |
| GOTERM_CC_3 | GO:0098803~respiratory chain complex | 1.60E-10 | 1.85E-07 |
| GOTERM_BP_3 | GO:1901564~organonitrogen compound metabolic process | 2.39E-05 | 0.03256821 |
| GOTERM_BP_3 | GO:1901657~glycosyl compound metabolic process | 8.53E-08 | 1.16E-04 |


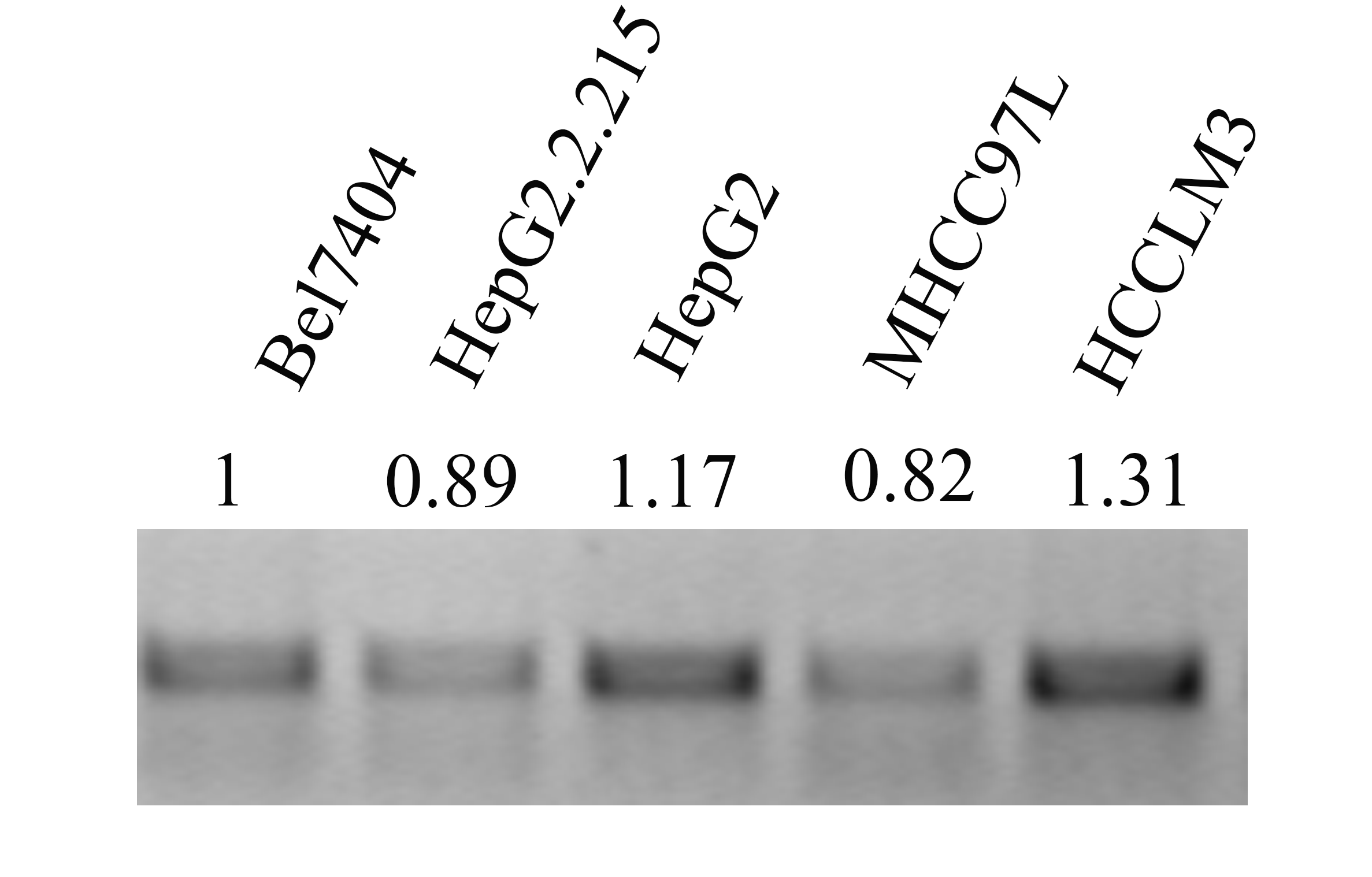


Supplementary Figure S1. Uncropped gel used in Fig. 1A.


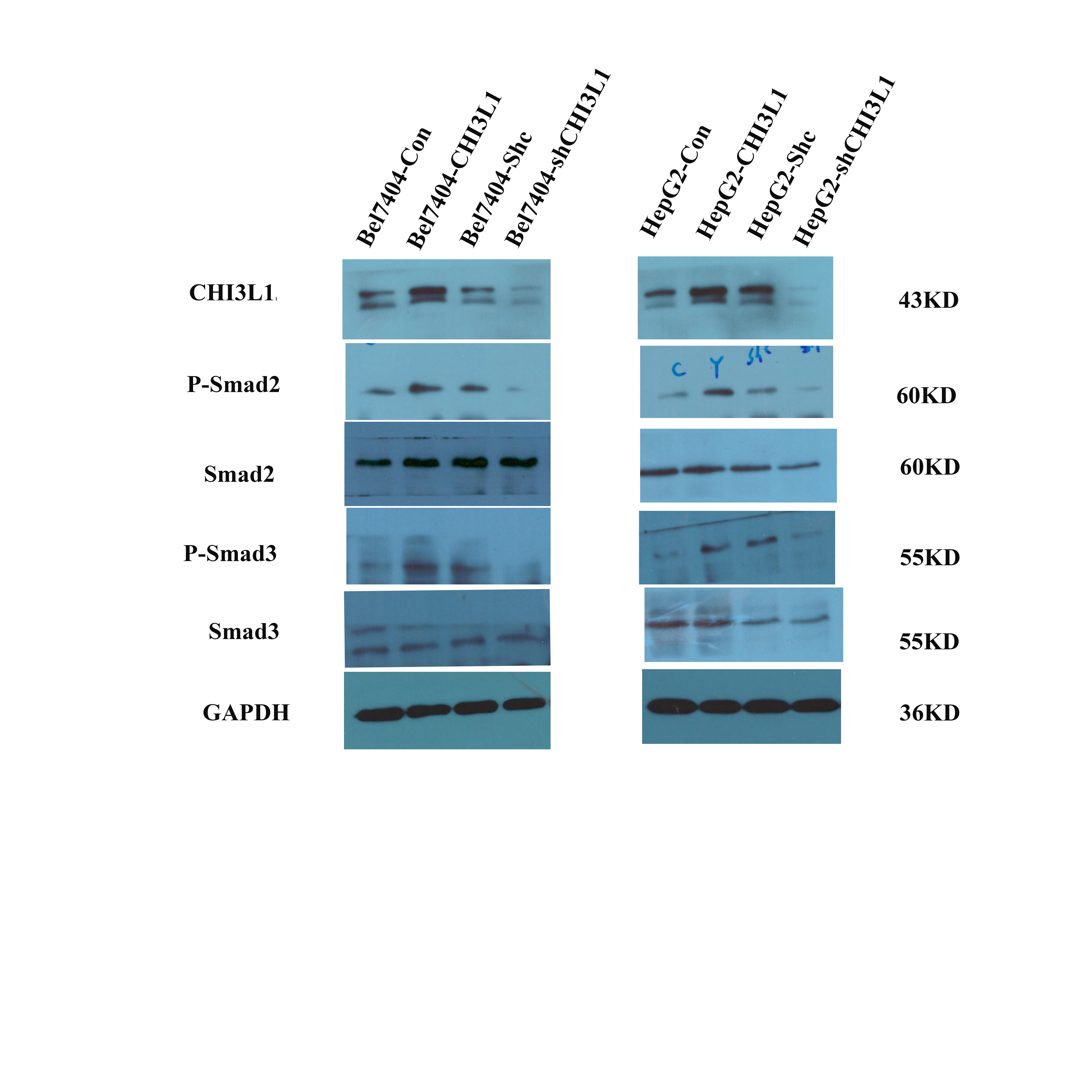


Supplementary Figure S2. Uncropped western blots used in Fig. 6A.


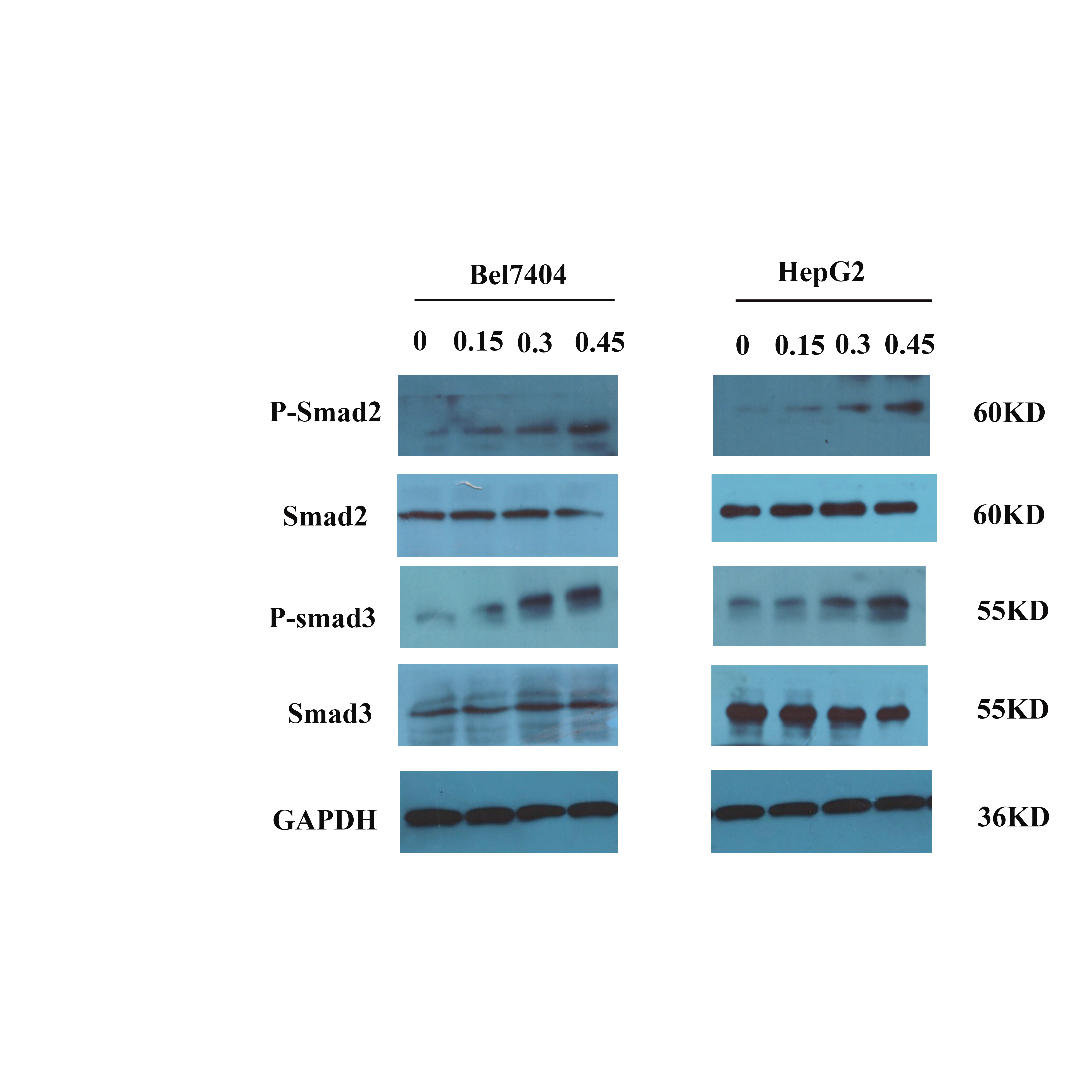


Supplementary Figure S3. Uncropped western blots used in Fig. 6E
